# *Momordica charantia* Suppresses Inflammation and Glycolysis in Lipopolysaccharide-Activated RAW264.7 Macrophages

**DOI:** 10.3390/molecules25173783

**Published:** 2020-08-20

**Authors:** Shi Yan Lee, Won Fen Wong, Jiyang Dong, Kian-Kai Cheng

**Affiliations:** 1 Innovation Centre in Agritechnology, Universiti Teknologi Malaysia, Pagoh 84600, Johor, Malaysia; leeshiyan0520@gmail.com; 2 Department of Medical Microbiology, Faculty of Medicine, University of Malaya, Kuala Lumpur 50603, Malaysia; wonfen@um.edu.my; 3 Department of Electronic Science, Xiamen University, Xiamen 361005, China; jydong@xmu.edu.cn

**Keywords:** bitter gourd, charantin, macrophage, NF-κB translocation, inflammation

## Abstract

Macrophage activation is a key event that triggers inflammatory response. The activation is accompanied by metabolic shift such as upregulated glucose metabolism. There are accumulating evidences showing the anti-inflammatory activity of *Momordica charantia*. However, the effects of *M. charantia* on inflammatory response and glucose metabolism in activated macrophages have not been fully established. The present study aimed to examine the effect of *M. charantia* in modulating lipopolysaccharide (LPS)-induced inflammation and perturbed glucose metabolism in RAW264.7 murine macrophages. The results showed that LPS-induced NF-κB (p65) nuclear translocation was inhibited by *M. charantia* treatment. In addition, *M. charantia* was found to reduce the expression of inflammatory genes including *IL6*, *TNF-*α, *IL1*β, *COX2*, *iNOS*, and *IL10* in LPS-treated macrophages. Furthermore, the data showed that *M. charantia* reduced the expression of *GLUT1* and *HK2* genes and lactate production (−28%), resulting in suppression of glycolysis. Notably, its effect on *GLUT1* gene expression was found to be independent of LPS-induced inflammation. A further experiment also indicated that the bioactivities of *M. charantia* may be attributed to its key bioactive compound, charantin. Taken together, the study provided supporting evidences showing the potential of *M. charantia* for the treatment of inflammatory disorders.

## 1. Introduction

Inflammation is a biological response of the host immune system to curb harmful stimuli [1], but dysregulation of inflammatory response has also been associated with chronic diseases, including stroke, heart disease, obesity, diabetes, arthritis, and cancer [2]. In a laboratory, inflammation is often studied using macrophages treated with lipopolysaccharide (LPS), a potent innate immune-activating stimuli which can directly activate macrophages [3]. Upon activation by LPS, RAW264.7 mouse macrophage cells showed increased production of measurable inflammatory mediators such as leukotrienes, tumor necrosis factor-alpha (TNF-α), and interleukins (ILs) [4].

The inflammatory response in activated macrophages has also been associated with profound reprogramming in cellular metabolism [5]. Previously, activated macrophages were found to preferentially metabolize glucose as an energy source and exhibit a Warburg-like shift in cellular metabolism [6]. The authors showed that the inflammatory capacity of activated macrophages is driven by GLUT1-mediated glucose uptake and enhanced glucose metabolism through the pentose phosphate pathway [6]. These findings suggested a link between inflammation and upregulated glucose metabolism.

There has been an increasing research interest on the anti-inflammatory effects of plant-based products such as *Momordica charantia* (MC) [7]. In a laboratory, wild MC was found to suppress LPS-stimulated inflammatory responses in macrophages through the inhibition of NF-κB activation [8]. In addition, the ethanol extract of MC was found to reduce LPS-induced nitric oxide, inducible nitric oxide synthase (*iNOS*), prostaglandin E2 (*PGE_2_*), and *pro-IL1*β expression [8]. It has also been shown that protein expression of COX2 and iNOS in activated mouse macrophages can be inhibited with an ethyl acetate extract of the MC fruit [9]. In addition, pre-treatment of the MC powder suppressed the expression of proinflammatory cytokines and inflammatory markers in cardiac tissue of rat models of myocardial infarction [10]. In another study, the administration of a freeze-dried MC powder significantly decreased the protein levels of NF-κB and JNK in a mouse model [11]. It has also been reported that MC supplementation reduces adipose tissue inflammation associated with macrophage infiltration in diet-induced obese mice [12].

Previously, a number of biological compounds in MC have been associated with its anti-inflammatory activity. The compounds include BG-4 [13], 5β,19-epoxy-25-methoxy-cucurbita-6,23-diene-3β,19-diol (EMCD, a triterpene) [14], and 3β,7β,25-trihydroxycucurbita-5,23(*E*)-dien-19-al (TCD) [15]. Charantin is one of the key bioactive compounds of MC. However, charantin is known for its anti-diabetic activity [16,17,18], and its anti-inflammatory potential has yet to be elucidated. The anti-inflammatory activity of MC shows potential pharmacological value, but the abilities of the plant extract to regulate metabolism and inflammatory events in macrophages are not fully understood. The present study provided further evidence on the anti-inflammatory activity of MC which may be linked with downregulation of the glycolytic pathway.

## 2. Results

### 2.1. Momordica charantia Suppresses Inflammation and Glycolytic Pathway

**Figure 1:**
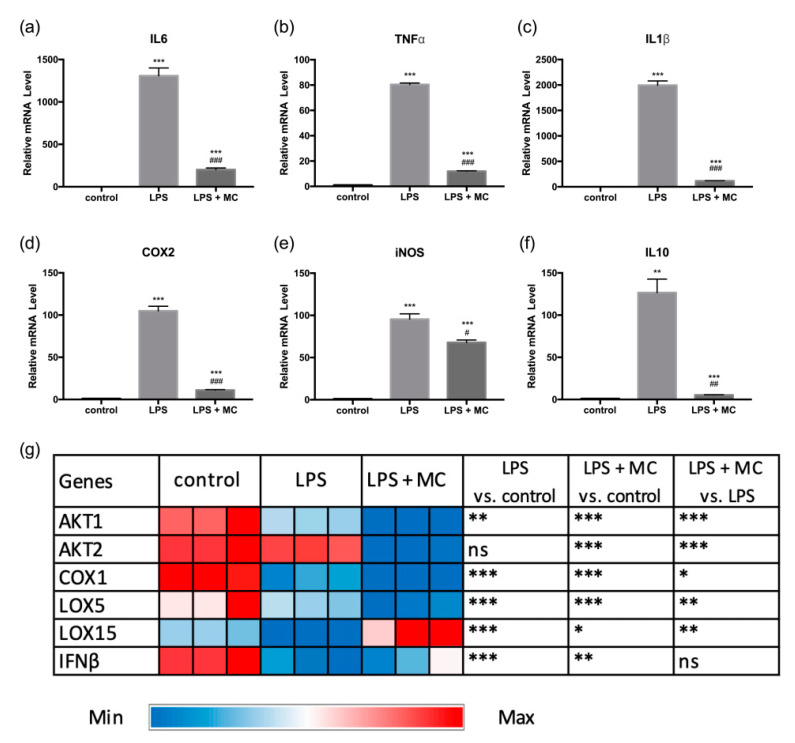
Effect of lipopolysaccharide (LPS) and *Momordica charantia* (MC) treatment on the expression of inflammatory genes: (**a**) *IL6*, (**b**) *TNF-*α, (**c**) *IL1*β, (**d**) *COX2*, (**e**) *iNOS*, and (**f**) *IL10*. *(***g**) Heatmap showing the expression of six selected genes including *AKT1*, *AKT2*, *COX1*, *LOX5*, *LOX15*, and *IFN*β. The RAW264.7 macrophages were pre-treated with MC water extract for four hours at the concentration of 12.5% *v*/*v* (equivalent to 5 mg/mL soluble MC extract), followed by incubation with 1 μg/mL LPS for four hours. The control group was treated with an equal volume of MiliQ water for a similar incubation time. For (**a**–**f**), the qRT-PCR data are expressed as mean ± SEM. Group comparison was performed using the Student’s *t*-test. For (**a**–**f**), ** *p* < 0.01, *** *p* < 0.001 compared with the control group; # *p* < 0.05, ## *p* < 0.01, ### *p* < 0.001 compared with the LPS group. For (**g**), * *p* < 0.05, ** *p* < 0.01, *** *p* < 0.001; ns: no significant difference between groups.

Firstly, the mRNA expression levels of 12 inflammatory genes were measured in RAW264.7 cells by real-time PCR. Following LPS stimulation, the mRNA levels of *IL6*, *TNF-*α, *IL1*β, *COX2*, *iNOS*, and *IL10* were strongly increased (Figure 1a–f), while the MC treatment (at a final concentration equivalent to 5 mg/mL soluble MC extract) reduced the expression levels of these genes by up to 84.7%, 85.1%, 94.2%, 89.5%, and 28.8%, respectively (Figure 1a–f). Previously, Hsu and colleagues showed that an ethyl acetate extract of MC suppressed mRNA expression of *IL6*, *TNF-*α, *COX2*, and *iNOS* induced by LPS in RAW264.7 cells [9]. In another study, Jones and colleagues reported that BG-4, a peptide with strong trypsin-inhibitory properties extracted from MC seeds reduces the production of IL6 and TNF-α in LPS-treated THP-1 macrophages [13]. Our results were consistent with these previous findings.

In addition, LPS treatment caused a marked 126-fold increase in anti-inflammatory IL10 gene expression (Figure 1f), consistent with findings reported by Pengal and colleagues [19]. This may be due to the cellular response to LPS-induced inflammation. Previously, Ip and colleagues showed that IL10 treatment exhibited anti-inflammatory activity by suppressing LPS-induced glucose uptake and glycolysis and promoted oxidative phosphorylation [20]. Following MC treatment, the IL10 gene expression was significantly suppressed, probably due to reduced inflammatory response in cells.

Furthermore, our data showed that the mRNA expression of *AKT1*, *COX1*, *LOX5*, *LOX15*, and *IFN*β were downregulated following LPS treatment (Figure 1g, Supplementary Figure S1). The expression of *AKT1*, *AKT2*, *COX1*, and *LOX5* genes was also reduced by the MC treatment. A previous study showed that LPS exposure suppressed LOX5 metabolism [21]. This may be due to the synthesis of NO by increased iNOS, as endogenous NO was found to inhibit lipoxygenase metabolism [22]. On the other hand, MC was found to upregulate the gene expression of LOX15, which may also lead to the production of anti-inflammatory metabolites [23]. Although LPS downregulated *COX1* gene, it was found to upregulate *COX2* gene expression up to 100-fold (Figure 1g, Supplementary Figure S1). A similar trend was reported in a previous study [24]. COX1 is constitutively expressed in most tissues, while COX2 is an inducible enzyme that responds to pro-inflammatory stimuli [24]. The effect of LPS on COX2 and COX1 may be accompanied by high production of PGE_2_, which may be prevented by a significant decrease in *COX2* gene expression following MC treatment.

### 2.2. Momordica charantia Inhibits NF-κB Translocation

**Figure 2:**
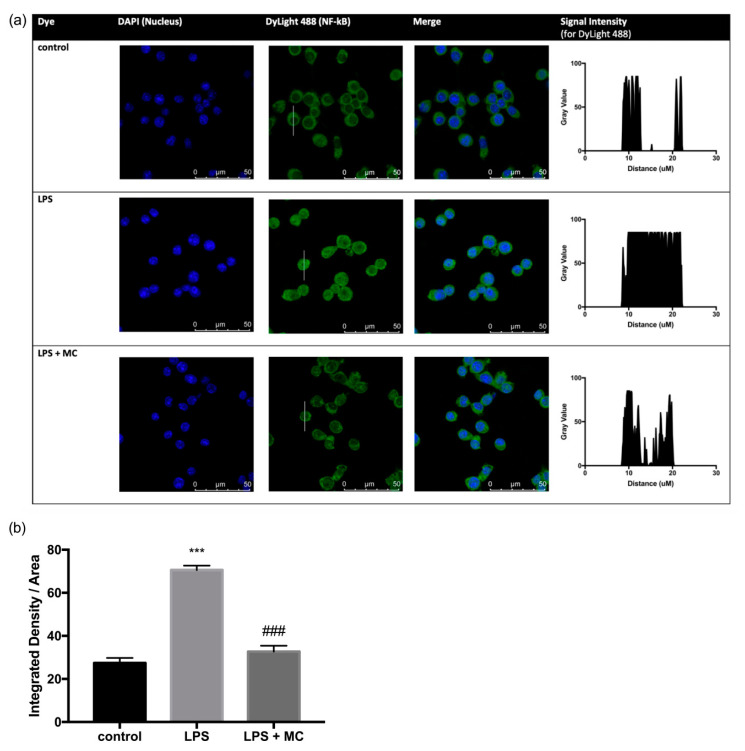
MC pre-treatment inhibited LPS-induced NF-κB translocation in RAW264.7 cells. (**a**) Immunofluorescence staining images for macrophages treated with or without LPS and MC. Right panel shows the gray value plot profile, which is representative of fluorescence intensities along the length of a drawn line shown in the middle panel, (**b**) Integrated density per area of cell, which represents the average intensity of protein immunostaining. In Figure 2a, the green dye 488 of control group showed a disk-like shape with a hollow center, suggesting that NF-κB was mainly present in cytoplasm. With LPS treatment, the 488 dye appeared brighter and the center of the cell was stained and in the merged image, the green dye and 4′,6-diamidino-2-phenylindole (DAPI) overlapped each other, implying the presence of NF-κB in the nucleus of the cell. In the treatment group, a hollow disk-like shape was observed with the stain of 488 dye, and the merged image showed that the DAPI-stained nucleus was surrounded with a ring of green dye, showing that NF-κB was mainly present in the cytoplasm. *** *p* < 0.001 compared with the control group. ### *p* < 0.001 compared with the LPS group.

To further investigate the role of NF-κB translocation in the anti-inflammatory effect of MC, we treated RAW264.7 cells with MC prior to LPS stimulation (a potent inducer and activator of NF-κB). NF-κB acts as a central mediator of inflammatory responses and compounds that inhibit NF-κB activation are potential anti-inflammatory agents. In the present study, we performed immunofluorescence staining of NF-κB p65. In the absence of LPS, we showed that NF-κB p65 remained in the cytoplasm of RAW264.7 cells (Figure 2a). In response to LPS stimulation, NF-κB p65 translocated from cytoplasm into the nucleus, implying NF-κB activation (Figure 2a). Our data showed that the average integrated density of DyLight-488 per area of cell treated with LPS was increased by 157% compared with the controls (Figure 2b). *M. charantia* was found to suppress the LPS-induced NF-κB translocation to a level comparable to that of the control RAW264.7 cells (Figure 2b).

### 2.3. Momordica charantia Suppresses Glucose Metabolism

**Figure 3:**
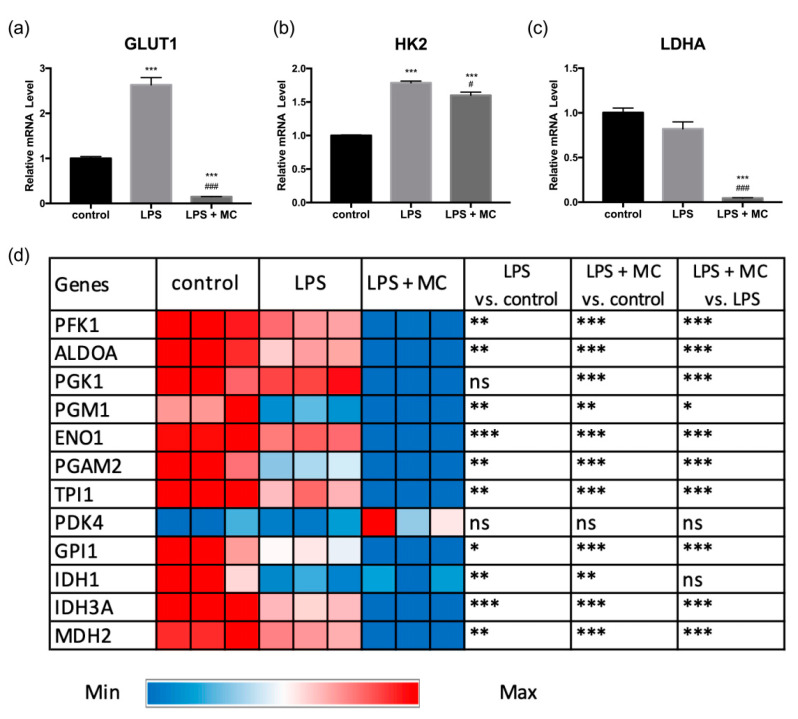
Effect of LPS and MC treatment on the expression of genes in glucose metabolism: (**a**) *GLUT1*, (**b**) *HK2*, and (**c**) *LDHA.* (**d**) Heatmap showing the expression of 12 selected genes including *PFK1*, *ALDOA*, *PGK1*, *PGM1*, *ENO1*, *PGAM2*, *TPI1*, *PDK4*, *GPI1*, *IDH1*, *IDH3A*, and *MDH2*. RAW264.7 macrophages were pre-treated with MC water extract for four hours, followed by incubation with 1 μg/mL LPS for four hours. The control group was treated with an equal volume of MiliQ water for a similar incubation time. The qRT-PCR data are expressed as mean ± SEM. Group comparison was performed using the Student’s *t*-test. For (**a**–**c**), *** *p* < 0.001 compared with the control group; # *p* < 0.05, ### *p* < 0.001 compared with the LPS group. For (**d**), * *p* < 0.05, ** *p* < 0.01, *** *p* < 0.001; ns, no significant difference between groups.

On the other hand, LPS and MC treatments were found to cause marked changes in the expression of genes in glucose metabolism. The notable changes included gene expression of *GLUT1* and *HK2*. Following LPS treatment, the expression of *GLUT1*, the main glucose transporter of macrophages, was increased by 163% (Figure 3a). The upregulated *GLUT1* gene expression by LPS was also previously reported by Kim and colleagues [25]. In addition, the gene expression of the rate-limiting enzyme for glycolysis, *HK2*, was also increased by 79% with LPS activation (Figure 3b). MC treatment markedly suppressed the gene expression of *GLUT1* and decreased *HK2* expression slightly in LPS-activated macrophages. Although LPS-mediated inflammation has been associated with increased glycolysis, no significant change was observed comparing *LDHA* gene expression between the controls and the LPS group (Figure 3c). However, the expression was significantly lower in the LPS + MC group. For the other studied genes in glycolysis and the tricarboxylic acid (TCA) cycle, there was a general trend by which the expression of genes (including *PFK1*, *ALDOA*, *PGM1*, *ENO1*, *PGAM2*, *TPI1*, *LDHA*, *GPI1*, *IDH3A*, and *MDH2*) was downregulated following LPS activation and the expression was further reduced with MC treatment (Figure 3d, Supplementary Figure S2).

**Figure 4:**
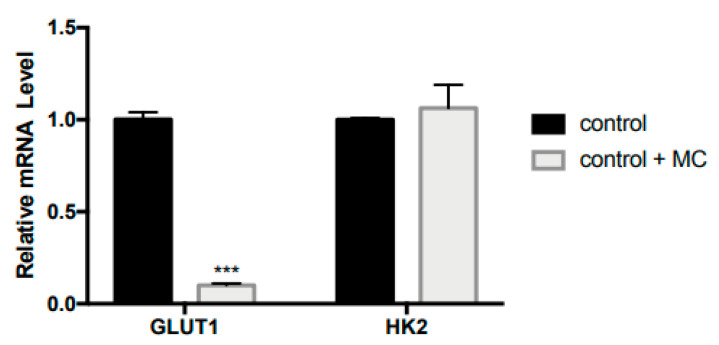
MC treatment reduced GLUT1 gene expression in macrophages in the absence of LPS-induced inflammation. Group comparison was performed using the Student’s *t*-test (*** *p* < 0.001).

Previously, an inflammatory event has been associated with enhanced glycolysis. Here, we found that only two out of 15 studied genes in glucose metabolism (i.e., *GLUT1* and *HK2*) showed upregulated expression following the LPS treatment. Thus, we conducted a further experiment by treating the control macrophage cells with MC to investigate the effect of MC on the expression of *GLUT1* and *HK2* genes in the absence of LPS-induced inflammation. The results showed that MC significantly reduced (*p* < 0.001) *GLUT1* (Figure 4), but not *HK2*, gene expression in the absence of LPS activation. The results indicated that the MC effect on *GLUT1* does not depend on the inflammatory event in macrophages. The present study suggested potential roles of *GLUT1* and glucose metabolism in LPS-induced inflammation events, and the potential bioactivity of MC in modulating glucose metabolism in RAW264.7 cells.

### 2.4. Momordica charantia Suppresses Lactate Production in LPS-Activated Macrophages

**Figure 5:**
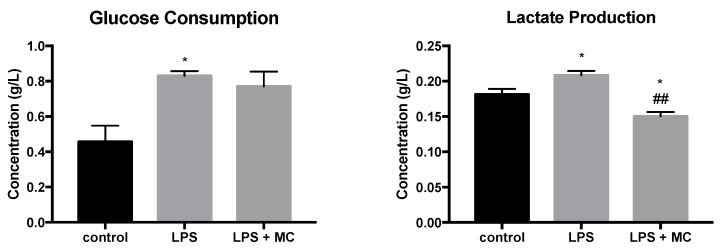
Effect of LPS and MC treatments on glucose consumption and lactate production. RAW264.7 macrophages were pre-treated with MC water extract for four hours at the concentration of 12.5% *v*/*v* (equivalent to 5 mg/mL soluble MC extract), followed by incubation with 1 μg/mL LPS for four hours. The control group was treated with an equal volume of MiliQ water for a similar incubation time. Data are expressed as mean ± SEM. Group comparison was performed using the Student’s *t*-test. * *p* < 0.05 compared with the control group. ## *p* < 0.01 compared with the LPS group.

To examine the effect of LPS stimulation and MC treatment in glucose utilization, the glucose and lactate concentrations in extracellular medium were examined. Our data showed that glucose consumption was significantly increased by 82% following LPS stimulation, but no significant difference was observed comparing the LPS groups with and without MC treatment (Figure 5). In addition, the production of lactate was higher (+15%) in LPS-stimulated RAW264.7 cells, suggesting upregulated glycolysis in the activated macrophages. Notably, MC treatment led to reduced lactate production (−28%), compared with the LPS group.

### 2.5. The Bioactivities of Momordica charantia May Be Attributed to Charantin

**Figure 6:**
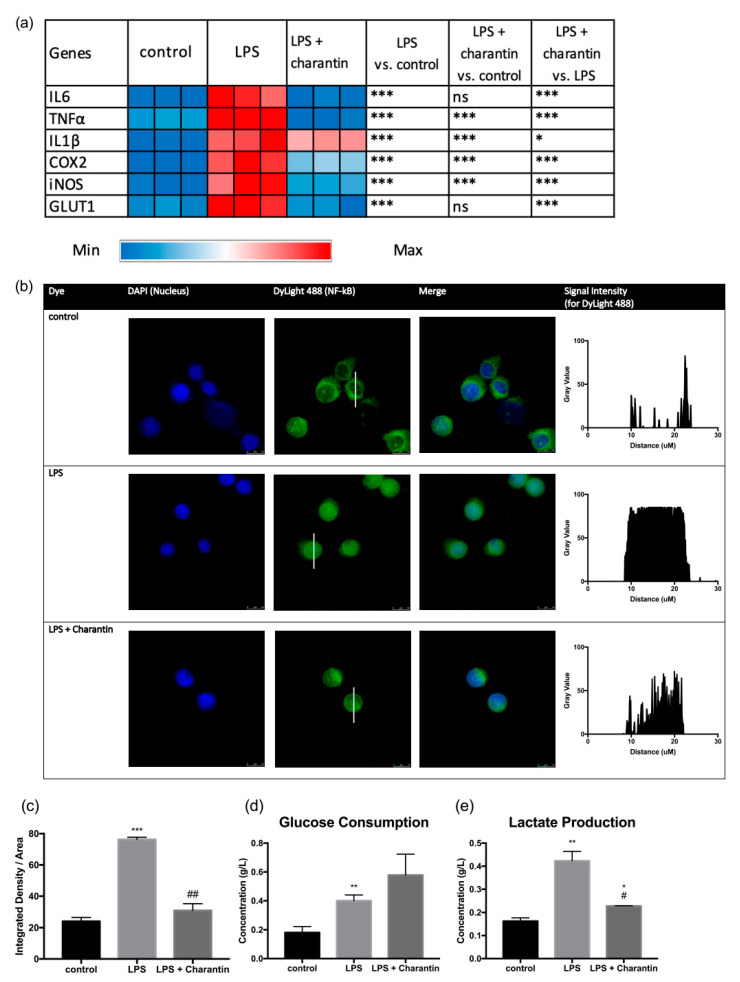
Bioactivities of charantin. (**a**) Charantin downregulated the expression of genes including *IL6*, *TNF-*α, *IL1*β, *COX2*, *iNOS*, and *GLUT1* in LPS-activated macrophages. (**b**) Charantin suppressed NF-κB translocation following LPS stimulation and (**c**) its corresponding integrated density/area plot. Effect of LPS and MC treatments on (**d**) glucose consumption and (**e**) lactate production. For (**a**), * *p* < 0.05, *** *p* < 0.001; ns, no significant difference between groups. For (**c**–**e**), data are presented as mean ± SEM. Group comparison was performed using the Student’s *t*-test. * *p* < 0.05, ** *p* < 0.01, *** *p* < 0.001 compared with the control group; # *p* < 0.05, ## *p* < 0.01 compared with the LPS group.

Next, we conducted a further experiment by treating the LPS-activated RAW264.7 cells with 10 µg/mL of charantin, a key bioactive compound in MC. The expression of six selected genes (including *IL6*, *TNF-*α, *IL1*β, *COX2*, *iNOS*, and *GLUT1*) were examined. All the studied genes were significantly downregulated following charantin treatment (Figure 6a; all *p* < 0.001, except *p* < 0.05 for *IL1*β), consistent with results from the MC experiment presented in Figure 1 and Figure 3.

Charantin treatment was found to inhibit NF-κB translocation (Figure 6b), consistent with the MC treatment. In addition, there was no significant difference in glucose consumption between the LPS and the LPS + charantin groups (Figure 6d). However, charantin treatment reduced lactate production by 46.2%, compared with the LPS group. Taken together, charantin was found to exert anti-inflammatory effects and inhibit glycolysis in LPS-activated macrophages. The data also suggested that the bioactivities of MC reported in this study may be attributed to charantin.

## 3. Discussion

In response to inflammatory stimulants, such as LPS, cells could mount immune responses through the activation of NF-κB, a key transcription factor that regulates innate and adaptive immune responses. NF-κB’s activity is mainly mediated by its nuclear translocation [26]. The results of the present study showed that LPS-induced phosphorylation and nuclear translocation of NF-κB may be inhibited in the presence of MC. The inhibitory effect of MC on NF-κB translocation was consistent with a previous finding where EMCD, a triterpene purified from MC, inhibited the activation of the IKK complex (an upstream signaling pathway of NF-κB pathway) and the NF-κB pathway, independent of AMP-activated protein kinase (AMPK) [14]. In addition, Jones and colleagues also suggested that MC reduced the phosphorylation of ERK 1/2 and STAT3 and thus dampened the NF-κB/MAPK/STAT3 signaling pathways, affecting the nuclear translocation of NF-κB [13]. Following LPS stimulation, the increased NF-κB transcription may upregulate GLUT1 surface localization [27,28] and *GLUT1* gene expression, leading to increased glycolysis as observed in the present study.

Upon stimulation with cytokines or LPS, resting macrophages shift their phenotype toward a pro-inflammatory state as part of the innate immune response. The LPS-activated macrophages undergo profound metabolic changes to adapt to these new physiological requirements. The reprogramming during macrophage activation shows overlapping features with cancer cells—both have increased glycolytic rates and increased lactate release, known as aerobic glycolysis or the Warburg effect [29].

In the present study, we observed transcriptional changes suggesting that the glycolytic pathway was upregulated in LPS-stimulated macrophages, as evidenced by increased glucose utilization, upregulation of mRNA expression of *GLUT1* and *HK2* (rate-limiting enzymes during glycolysis), and increased production of lactate. Previous literature also reported that RAW264.7 cells increase glucose uptake by two- to three-folds in the presence of LPS [25,30]. A clinical study involving healthy individuals also reported that LPS administration led to consistent increases in plasma lactate concentration, suggesting that LPS stimulates lactate production, and the increased lactate concentration was found to not originate from skeletal muscle cells [31]. The potential role of glycolysis in the inflammatory response of macrophages has also been reported in a previous study, where inhibition of glycolysis using 2-deoxyglucose decreased LPS-induced IL1β expression [32]. In addition, overexpressed GLUT1 in activated macrophages was found to induce a proinflammatory response that was dependent on glycolysis and reactive oxygen species [6]. A glucose tracer-based metabolomics approach revealed that the activation of murine peritoneal macrophages through various toll-like receptor (TLR) pathways resulted in a highly glycolytic phenotype [33]. In another study, M2 (alternatively activated) macrophages displayed enhanced mitochondrial oxidative phosphorylation, whereas M1 (classically activated) macrophages predominantly used glycolysis to generate ATP [34]. A study on humans also presented results consistent with the observations in mice. Leukocytes of sepsis patients were found to undergo metabolic shift, which preferentially utilized aerobic glycolysis as the energy source, and this observation was reversed upon patient recovery [35].

**Figure 7:**
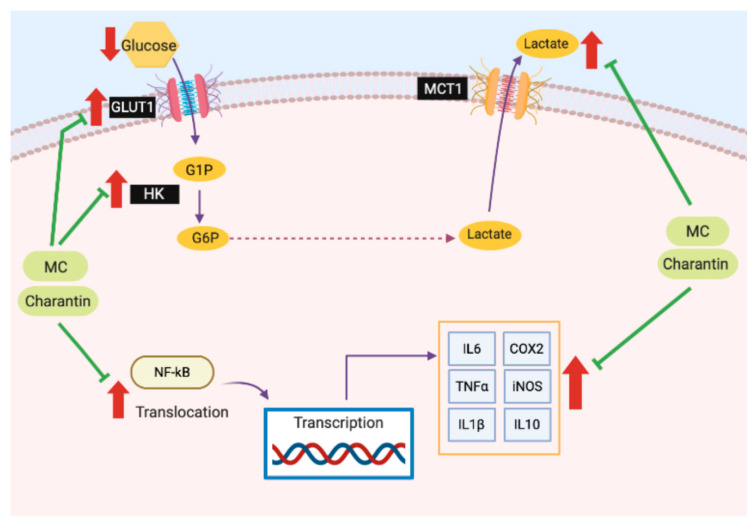
MC suppresses LPS-induced inflammation and perturbation in glucose metabolism, particularly on *GLUT1* and *HK* gene expression, and extracellular concentrations of glucose and lactate. The red arrows show the upregulation of mRNA expression of the selected genes and changes in concentration of metabolites induced by LPS treatment.

The present study suggested that the anti-inflammatory effect of MC may be linked with the downregulation of glucose metabolism (Figure 7). MC treatment attenuated the LPS-induced Warburg effect by reducing the gene expression of *GLUT1* and *HK2*. Notably, the *GLUT1* gene expression was also reduced when the controls were treated with MC, indicating that the metabolic effect of MC on *GLUT1* gene expression may not depend on the inflammatory event in cells. In an earlier literature study, MC was reported to reduce hexokinase activity and glucose uptake activity in intestinal fragments of rats [36]. Even though the other glycolytic genes were found to be slightly downregulated in the LPS group, the end-point glucose and lactate analysis indicated that the rate-limiting components, expression of *GLUT1* and *HK2* genes, may be the key factors for enhanced glycolysis due to inflammation. The inhibition of glucose metabolism offers potential clinical value in facilitating disease treatment. Previously, inhibition of PGK was reported to be effective in the treatment of cardiovascular and respiratory disorders [37]. Furthermore, the knockdown of ALDO exerted a suppressive effect on cell proliferation in *ras*-transformed NIH-3T3 cells, indicating that ALDO is a potential target in cancer treatment [38]. The present findings also suggested that inhibition of GLUT1 activity or its expression may serve as a therapeutic strategy for inflammation.

A number of bioactive compounds in MC have been associated with its anti-inflammatory activity. Previously, linolenoyl and linoleoyl types of lysophosphatidylcholines (LPCs) in MC were found to suppress LPS-induced inflammatory responses in RAW 264.7 cells by inhibiting the activation of MAPKs and NF-κB DNA-binding activity [39]. In addition, TCA, a biological compound in MC was shown to suppress the AKT/NF-κB pathway, leading to reduced proliferation of breast cancer cells [15]. Here, we showed that the anti-inflammatory activity of MC may be attributed to charantin, a key bioactive compound in MC known for its anti-diabetic property. Taken together, our study may provide new insights into the potential of MC and charantin for the treatment of inflammatory disorders.

## 4. Materials and Methods

### 4.1. Preparation of MC Extract

MC fruits were sourced from a local distributor located in Johor, Malaysia. A voucher specimen identified as *Momordica charantia* L. (Voucher No. UNMC 5600/19) was deposited in the herbarium of the University of Nottingham Malaysia. For sample preparation, the fruits were first cleaned and cut into 5 mm thin pieces and kept in a freezer at −20 °C. Then, the frozen fruits were freeze-dried using the Cuddon FD80 freeze-dryer and subsequently ground into a fine powder. The MC powder was then added to MiliQ water in 1:9 *w*/*w* ratio (MC:water). The solution was vortex-mixed and the resulting aqueous MC extract was filtered using a 3 kDa spin column before the cell culture experiment.

### 4.2. Cell Culture Experiment

RAW264.7 cells were purchased from ATCC (TIB71) and were cultured in Dulbecco’s Modified Eagle Medium (DMEM; Gibco, Thermo Fisher Scientific, Waltham, MA, USA) supplemented with 10% heat-inactivated fetal bovine serum, penicillin G (100 U/mL), and streptomycin (100 μg/mL). Cells were maintained at 37 °C in 5% CO_2_ in a humidified incubator [11].

For the cell proliferation assay, a total number of 1.0 × 10^4^ cells per well were seeded into a 96-well plate and incubated overnight at 37 °C in 5% CO_2_ to allow cell adherence to the plate. The next day, the cells were treated with MC water extracts at different concentrations. After 24 h, MTT (4,5-dimethylthiazol-2-yl-2,5-diphenyltetrazoliumbromide) reagent at a final concentration of 50 μg/mL was added. After three hours of incubation at 37 °C in 5% CO_2_, the solution was removed. DMSO was then added to dissolve the formazan crystals. The plates were then read using the Synergy HTX multi-mode reader (BioTek Instruments, Winooski, VT, USA) at 570 nm. The ratio of the absorbance of treated cells to the absorbance of DMSO-treated control cells was determined as the percentage of cell viability. The concentration of compounds which resulted in a 50% reduction in cell viability was defined as IC_50_. The IC_50_ was found to be 30.48% *v/v* of MC extract volume per volume of culture medium (Supplementary Figure S3). Due to potential toxicity of the MC water extract [7], the incubation time for subsequent tests was designed to be not longer than 8 h to ensure that the cell number between each groups were comparable.

### 4.3. Gene Expression Analysis

Cells in T25 flask with 80% confluency pre-treated with or without 12.5% *v/v* MC water extract (final concentration equivalent to 5 mg/mL soluble MC extract) for four hours were incubated with 1 μg/mL LPS for four hours [40]. The total treatment time was 8 h and it was adequate to see changes in mRNA levels [41]. Total RNAs were extracted using TRIzol (Invitrogen, Waltham, MA, USA). Complimentary DNAs were synthesized using SuperScript IV kits (Invitrogen) according to the protocol provided by the manufacturer. The mixture of 20 μL in total volume was incubated in the Veriti Thermal cycler (Thermo Fisher Scientific, Waltham, MA, USA) with the setting of 50 °C for 10 min, followed by 80 °C for 10 min. Agarose gel electrophoresis and nanodrop were used to check the quality of the cDNA. Gene expression was quantified using real-time PCR with SYBR Green PCR Master mix (Thermo Fisher Scientific, Waltham, MA, USA) in an Agilent Stratagene Mx3000P real-time PCR system (Agilent, Waldbronn, Germany). Gene expression was normalized to β-*actin* mRNA level before further analysis. The selected genes included genes in the inflammatory pathway and genes involved in energy metabolism. The primer sequences of the related genes used in real-time PCR are provided as supplementary data (Supplementary Table S1).

### 4.4. Immunofluorescence Staining

A total number of 7.0 × 10^4^ cells per well were seeded into chamber slides and incubated overnight at 37 °C in 5% CO_2_ to allow attachment of the cells. Cells were pre-treated with or without the MC extract for four hours prior to stimulation with LPS for one hour due to the rapid reaction of macrophage cells toward LPS stimulation [40]. Cells were then fixed with ice-cold methanol for 10 min. The fixing solution was washed-off with a washing buffer (0.01% Triton X) three times for five minutes each. The cells were then incubated in 3% bovine serum albumin (BSA) in 0.1% Triton X buffer to block non-specific binding at room temperature for one hour, followed by a primary NF-κB p65 antibody (Cell Signaling Technology, Danvers, MA, USA) overnight at 4 °C. In the next day, the slides were washed before probing them with a DyLight 488-conjugated secondary antibody (Cell Signaling Technology, Danvers, MA, USA)) for one hour at room temperature in the dark. After that, the slides were washed again, followed by mounting the slides with cover slips and DAPI dye. The plate with stained cells was examined and images were captured using a Leica confocal laser scanning microscope (Leica Microsystem, Wetzlar, Germany).

For semi-quantitative analysis, the obtained cell images were examined using ImageJ 1.33u software (National Institutes of Health, Bethesda, MD, USA) to generate a gray value plot profile (representative of fluorescence intensities along the length of a drawn line) and an integrated density per area of cell plot, which represented the average intensity of protein immunostaining in five selected cells.

### 4.5. Glucose and Lactic Acid Analysis

A total number of 5.0 × 10^4^ cells per well were seeded onto a 48-well plate and incubated overnight at 37 °C in 5% CO_2_. The next day, the cells were treated with or without 12.5% *v*/*v* MC extract (final concentration equivalent to 5 mg/mL soluble MC extract) for four hours and 1 μg/mL LPS for four hours. The spent medium was collected and the analysis of glucose and lactic acid concentrations was determined using R-Biopharm D-Glucose kits (R-Biopharm AG, Darmstadt, Germany) and R-Biopharm L-Lactic acid kits (R-Biopharm AG, Darmstadt, Germany), respectively, according to the user manual provided by the manufacturer.

### 4.6. Bioactivities of Charantin

A further experiment was conducted to examine the bioactivities of charantin, a key bioactive compound in *M. charantia*. Charantin is a 1:1 mixture of two compounds, namely, sitosterol glucoside and stigmasterol glucoside. In our study, sitosterol glucoside (Chromadex, Irvine, CA, USA) and stigmasterol glucoside (ChemFaces, Wuhan, Hubei, China) were dissolved using DMSO:methanol (1:1) and methanol (Sigma Aldrich, St. Louis, MO, USA), respectively. The two compounds were then mixed in 1:1 ratio and filtered through a 0.2 µm syringe filter before further experiment.

A cell viability assay was conducted with the concentration of charantin ranging from 0 to 20 µg/mL (Supplementary Figure S4). From the result, charantin at a concentration of 10 µg/mL was selected for further experiment. Then, a cell culture experiment was carried out as described in Section 4.2. In addition, a gene expression analysis for six selected genes (including *IL6*, *TNF-*α, *IL1*β, *COX2*, *iNOS*, and *GLUT1*) was conducted as described in Section 4.3. Furthermore, an experiment on immunofluorescence staining and quantification of glucose and lactic acid was carried out as depicted in Section 4.4 and Section 4.5, respectively.

### 4.7. Statistical Analysis

All experiments were conducted in triplicates and repeated at least twice with consistent results. All the data were analyzed using Student’s *t*-test and the errors were presented as standard error of mean (SEM).

## 5. Conclusions

In this study, we provided evidence showing the anti-inflammatory activity of MC and its metabolic effect leading to downregulation of glycolysis in macrophages. MC effectively suppressed the mRNA expression of GLUT1 and glycolytic and inflammatory genes, and inhibited enhanced glycolysis in LPS-activated macrophages. These findings contribute to accumulating evidence suggesting the potential of MC in providing a therapeutic effect to inflammation and inflammation-related disorders.

## Supplementary Materials

The following are available online at, A Supplementary Data file which contains four figures and a table: Figure S1: Effect of LPS and MC treatment on the expression of selected inflammatory genes, Figure S2: Effect of LPS and MC treatment on the expression of selected genes in glucose metabolism, Figure S3: Cell viability assay of RAW264.7 cells treated with *Momordica charantia* extract, Figure S4: Cell viability assay of RAW264.7 cells treated with charantin, Supplementary Table S1: List of primer sequences used in RT-PCR.
