# Supplementary material for: Momordica charantia Suppresses Inflammation and Glycolysis in Lipopolysaccharide-Activated RAW264.7 Macrophages
Source: Molecules. 2020 Aug 20;25(17):3783. doi: 10.3390/molecules25173783 (PMC7504525; doi:10.3390/molecules25173783)
Supplement: Supplementary file 1 [file molecules-25-03783-s001.pdf]

SUPPLEMENTARY MATERIALS

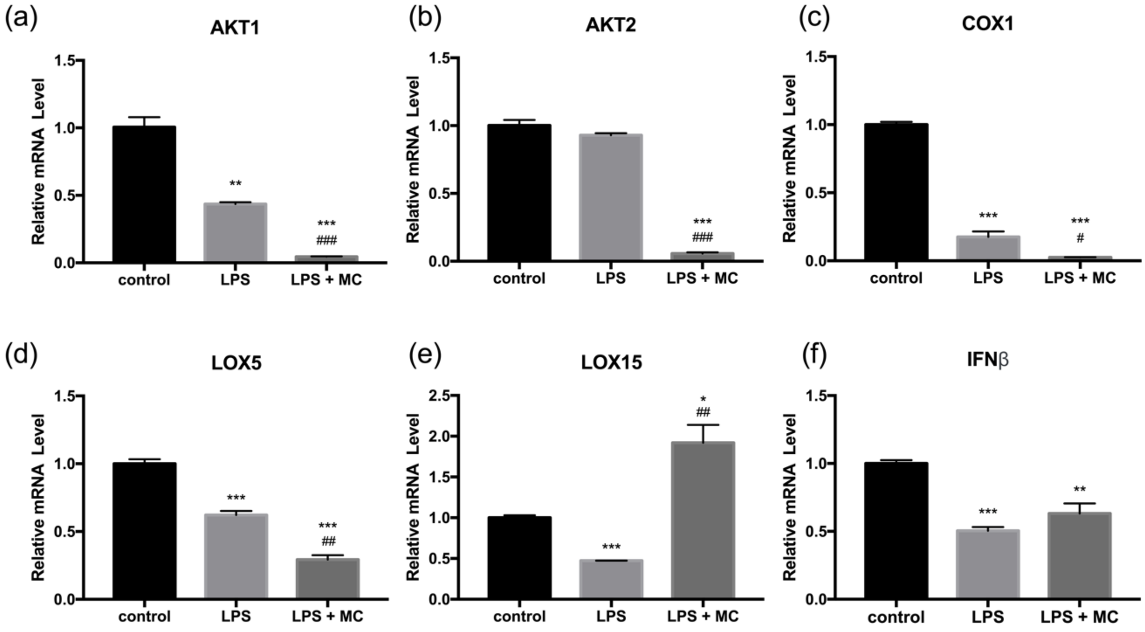

Supplementary Figure S1. Effect of LPS and MC treatment on the expression of selected inflammatory genes, including *AKT1, AKT2, COX1, LOX5, LOX15, and IFN*β. The data is also presented as a heatmap in Figure 1(**g**). The qRT-PCR data are expressed as mean ± SEM. Group comparison was performed using the Student's t test: * p<0.05, ** p<0.01, *** p<0.001 compared with the control group. # p<0.05, ## p<0.01, ### p<0.001 compared with the LPS group.

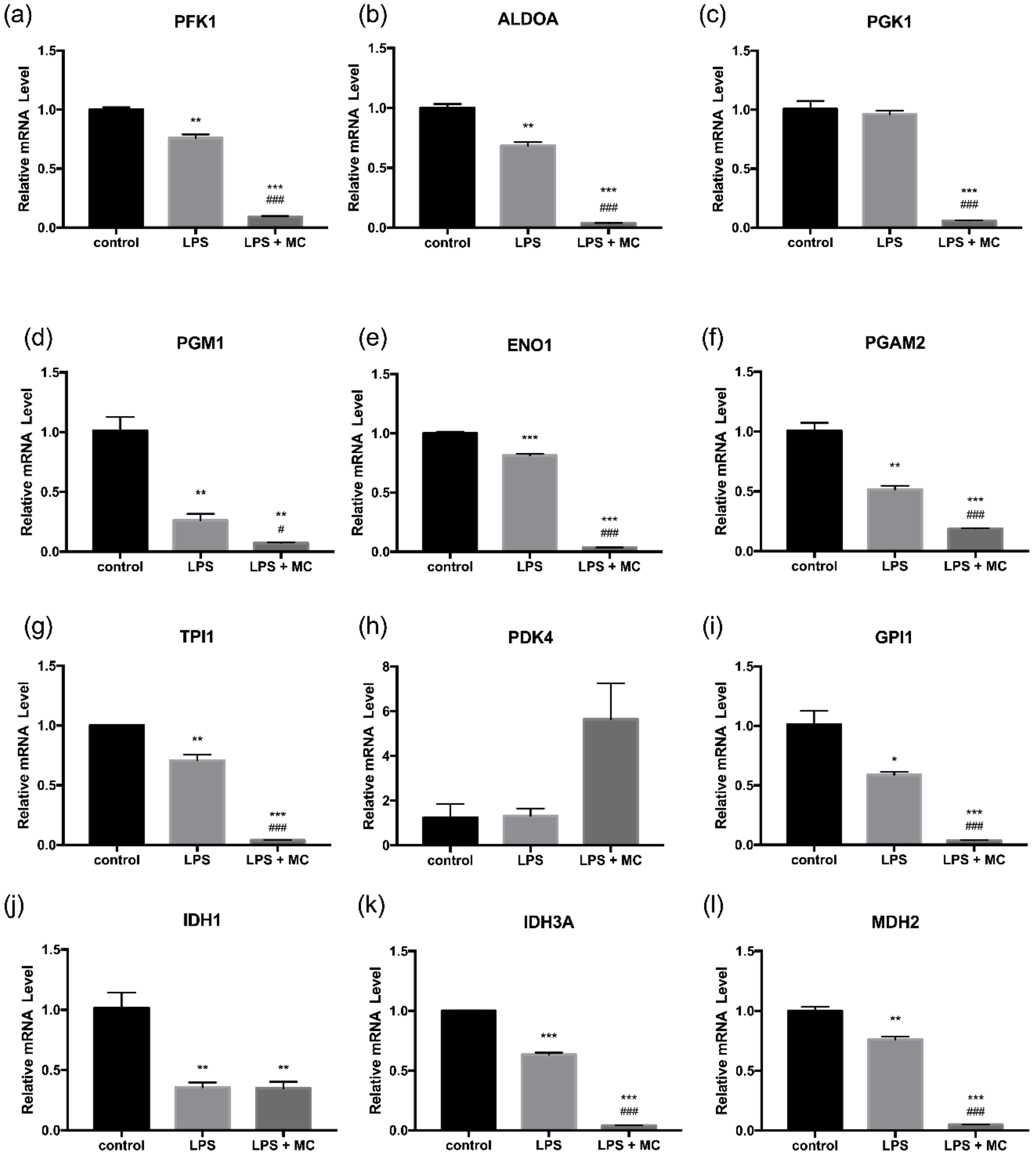

Supplementary Figure S2. Effect of LPS and MC treatment on expression of selected genes in glucose metabolism, including *PFK1*, *ALDOA*, *PGK1*, *PGM1*, *ENO1*, *PGAM2*, *TPI1*, *PDK4*, *GPI1*, *IDH1*, *IDH3A*, and *MDH2*. The data is also presented as a heatmap in Figure 3(**d**). The qRT-PCR data are expressed as mean ± SEM. Group comparison was performed using the Student's t test: * $p < 0.05$, ** $p < 0.01$, *** $p < 0.001$ compared with the control group. # $p < 0.05$, ### $p < 0.001$ compared with the LPS group.

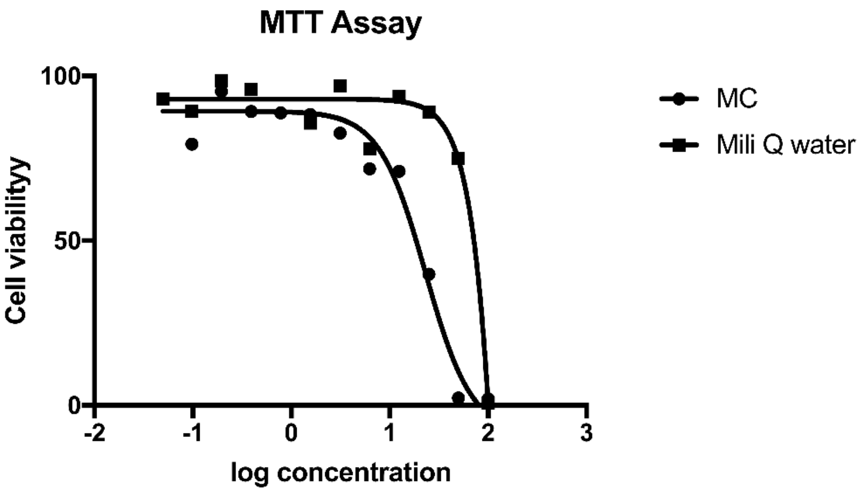

Supplementary Figure S3. Cell viability assay of RAW264.7 treated with *Momordica charantia* extract.

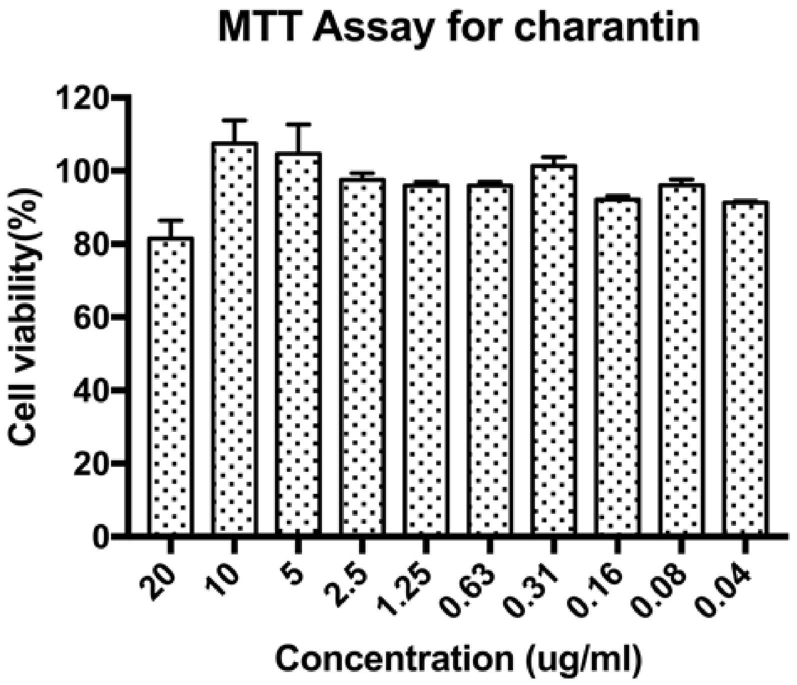

Supplementary Figure S4. Cell viability assay of RAW264.7 treated with charantin.

Supplementary Table S1. List of primer sequences used in RT-PCR.

Gene	Primer	Sequence (5' to 3')
		**Inflammatory Genes**
**IL-6**	Forward	TTCCCTACTTCACAAGTC
	Reverse	ACTAGGTTTGCCGAGTA
**IL-10**	Forward	GACGCTGTCATCGATTTCTCC
	Reverse	AGTAGATGCCGGGTGGTTCA
**TNF-*α***	Forward	TGCCTATGTCTCAGCCTCTTC
	Reverse	GAGGCCATTTGGGAACTTCT
**IL-1β**	Forward	GTTGACGGACCCCAAAAGAT
	Reverse	CCTCATCCTGGAAGGTCCAC
**IFN-g**	Forward	TCCAAGAAAGGACGAACATTCG
	Reverse	GAGGCCATTTGGGAACTTCT

**iNOS**	Forward	GTCTTGGTGAAAGTGGTGTT
	Reverse	GTGCTTGCCTTATACTGGTC
**AKT1**	Forward	GTCTCTAGGGTCCAGGGCCAAAGTC
	Reverse	CATCTAAAAGGACAAGTGCTAGGAG
**AKT2**	Forward	CCAAGACAGTATTGGGCCCCTTGGA
	Reverse	AGCCTGGGATGCTCACTGCTAGGTC
**COX1**	Forward	AGGAGATGGCTGCTGAGTTGG
	Reverse	AATCTGACTTTCTGAGTTGCC
**COX2**	Forward	ATCTGGCTTCGGGAGCACAAC
	Reverse	GAGGCAATGCGGTTCTGATACTG
**LOX5**	Forward	TCTGGTGTCTGAGGTGTTCG
	Reverse	AACCTCACATGGGCTACCAG
**LOX15**	Forward	AGCTCATTGTGTCCCCCTGAT
	Reverse	ACATTCCCACCACGTACCGAT
	**Genes involved in glucose metabolism**	
**GLUT1**	Forward	CATCGCCCTGGCCCTGCAGGAGC
	Reverse	GGCACCCCCCTGCCGGAAGCCGGA
**HK2**	Forward	TGATCGCCTGCTTATTCACGG
	Reverse	AACCGCCTAGAAATCTCCAGA
**PFK1**	Forward	GGGTCATGTACAGCGAGGA
	Reverse	GGCCTCCATACCCATCTTG
**ALDOA**	Forward	CAGGAAAGCAACTGCCACCGGCAC
	Reverse	GGATTCACACGGTCGTCTGCAGTC
**GAPDH**	Forward	AAGGTCATCCCAGAGCTGAA
	Reverse	ATGTAGGCCATGAGGTCCAC
**PGK1**	Forward	AACCTCCGCTTTCATGTAGAG
	Reverse	GACATCTCCTAGTTTGGACAGTG
**PGM1**	Forward	AACAAGATGCCCTTGGGAGCTGTGA
	Reverse	GAACTGATTGGACAGAAGGCACTAG
**ENO1**	Forward	CGCGTCTGTCCTTAAGGCTCTC
	Reverse	GCGGTGTACAGATCGACCTCA
**PGAM2**	Forward	GCTGCCACCTAGAGTTCCTG
	Reverse	GGGCTGCAATAAGCACTCTC
**TPI1**	Forward	TGACCTTCAGAGACTTGAGCC
	Reverse	CGGTGGGAGCAGTTACTAAAC
**LDHA**	Forward	GCTCCCCAGAACAAGATTACAG
	Reverse	TCGCCCTTGAGTTTGTCTTC
**PDK4**	Forward	GAGGATTACTGACCGCCTCTTTAG
	Reverse	TTCCGGGAATTGTCCATCAC
**GPI1**	Forward	CAGAGACAGCAAAGGAGTGG
	Reverse	GTAGACAGGGCGACAAAGTG
**IDH1**	Forward	ATGTCCAAAAAAATCAGTGGCGGTTCT
	Reverse	TTAAAGTTTGGCCTGAGCTAGTTTGATC
**IDH3A**	Forward	TGGGTGTCCAAGGTCTCTC
	Reverse	CTCCCACTGAATAGGTGCTTTG
**MDH2**	Forward	TTCAACACCAACGCTACCATTGTG
	Reverse	GTGTTCGCTCTGACGATG TCAAGG
	**General Gene**	
**β-actin**	Forward	GATTACTGCTCTGGCTCCTAGC
	Reverse	ACTCATCGTA CTCC TGCTTGCT
